# Usability, Acceptability, and Safety Analysis of a Computer-Tailored Web-Based Exercise Intervention (ExerciseGuide) for Individuals With Metastatic Prostate Cancer: Multi-Methods Laboratory-Based Study

**DOI:** 10.2196/28370

**Published:** 2021-07-28

**Authors:** Holly EL Evans, Cynthia C Forbes, Daniel A Galvão, Corneel Vandelanotte, Robert U Newton, Gary Wittert, Suzanne Chambers, Andrew D Vincent, Ganessan Kichenadasse, Danielle Girard, Nicholas Brook, Camille E Short

**Affiliations:** 1 Freemasons Centre for Male Health & Wellbeing School of Medicine University of Adelaide Adelaide Australia; 2 Wolfson Palliative Care Research Centre Institute of Clinical and Applied Health Research Hull York Medical School, University of Hull Hull United Kingdom; 3 Exercise Medicine Research Institute Edith Cowan University Joondalup Australia; 4 Physical Activity Research Group Appleton Institute Central Queensland University North Rockhampton Australia; 5 School of Human Movement and Nutrition Sciences The University of Queensland Brisbane Australia; 6 Faculty of Health Sciences Australian Catholic University Brisbane Australia; 7 College of Medicine and Public Health Flinders Centre for Innovation in Cancer Bedford Park Australia; 8 Alliance for Research in Exercise, Nutrition and Activity Allied Health and Human Performance University of South Australia Adelaide Australia; 9 Department of Surgery School of Medicine University of Adelaide Adelaide Australia; 10 Melbourne Centre for Behaviour Change Melbourne School of Psychological Sciences The University of Melbourne Melbourne Australia; 11 Melbourne School of Health Sciences The University of Melbourne Melbourne Australia

**Keywords:** exercise, metastatic prostate cancer, behavioral change, eHealth, computer-tailoring, usability, acceptability

## Abstract

**Background:**

Digital health interventions such as tailored websites are emerging as valuable tools to provide individualized exercise and behavioral change information for individuals diagnosed with cancer.

**Objective:**

The aim of this study is to investigate and iteratively refine the acceptability and usability of a web-based exercise intervention (*ExerciseGuide*) for men with metastatic prostate cancer and determine how well individuals can replicate the video-based exercise prescription.

**Methods:**

A laboratory-based multi-methods design was used, incorporating questionnaires, think-aloud tests, interviews, and movement screening among 11 men aged 63 to 82 years with metastatic prostate cancer. Overall, 9 participants were undergoing androgen deprivation therapy, and 2 were completing chemotherapy. Data were collected in two waves, with changes made for quality improvement after participant 5.

**Results:**

The intervention’s usability score was deemed moderate overall but improved after modifications (from 60, SD 2.9 to 69.6, SD 2.2 out of 100). Overall, the participants found the intervention acceptable, with scores improving from wave 1 (24.2, SD 1.1 out of 30) to wave 2 (26.3, SD 2.1 out of 30). The personalized multimodal exercise prescription and computer-tailored education were seen as valuable. After wave 1, website navigation videos were added, medical terminology was simplified, and a telehealth component was included after expert real-time telehealth support was requested. Wave 2 changes included the added variety for aerobic exercise modes, reduced computer-tailoring question loads, and improved consistency of style and grammar. Finally, the participants could replicate the resistance exercise videos to a satisfactory level as judged by the movement screen; however, additional technique cueing within the videos is recommended to address safety concerns.

**Conclusions:**

The acceptability and usability of *ExerciseGuide* were deemed satisfactory. Various problems were identified and resolved. Notably, the participants requested the inclusion of personalized expert support through telehealth. The resistance training algorithms were shown to provide appropriate content safely, and the users could replicate the exercise technique unaided to a satisfactory level. This study has optimized the *ExerciseGuide* intervention for further investigation in this population.

**Trial Registration:**

Australian New Zealand Clinical Trials Registry (ANZCTR) ACTRN12618001978257; https://anzctr.org.au/Trial/Registration/TrialReview.aspx?ACTRN=12618001978257

## Introduction

### Background

Prostate cancer is the most prevalent cancer type and the second most common cause of cancer-related deaths among Australian men [1]. The 5-year survival rate for prostate cancer diagnosed at stage 1 (localized cancer) is 95% [2]. In contrast, the survival rate for stage 4 cancers (cancer metastasized beyond the tissues directly adjacent to the prostate gland) is just 36.4% [2]. However, therapeutic advances in the management of metastatic prostate cancer continue to extend survival time, necessitating a focus on supportive care to optimize quality of life, maintain function, and further improve the survival rate [3,4]. For example, individuals living with metastatic prostate cancer often present with numerous physical and psychological concerns, including cancer-related fatigue, urinary incontinence, pain, increased fat mass, reduced muscle mass, anxiety, and depression [4].

It has been well established that multimodal exercise (an intervention based on the combination of physical exercises of different components, such as cardiorespiratory and muscular strength) has been shown to maintain or improve well-being and physical functioning, including among men with localized prostate cancer [5]. However, until recently, exercise interventions were avoided for many individuals diagnosed with metastatic prostate cancer, particularly those with bone lesions, for fear of adverse events. Recent studies, including those by Galvao et al [6] and Cormie et al [7], have demonstrated the safety and preliminary efficacy of individually tailored, modular (designed to avoid excessive loading of lesion sites), and clinic-based exercise programs using randomized controlled trials, thus indicating that individually tailored exercise may provide a powerful addition to improve supportive care in this population.

Currently, individually tailored supervised exercise interventions delivered by oncology-trained exercise professionals are not extensively available outside of urban areas [6,8,9]. The time-related demands and financial pressures faced by men with metastatic prostate cancer may lead to reluctance or inability to attend supervised clinic-based exercise programs [4,10]. Recently, Brown et al [11] commenced research into a home-based exercise approach for individuals with metastatic prostate cancer, which uses a one-time face-to-face exercise assessment, print-based material, and weekly telephone contact for remote supervision and behavioral change counseling. To further increase the scalability, accessibility, and adherence to home-based exercise, the addition of digital technologies to this type of home-based exercise intervention may be advantageous.

One type of digital technology that could be a viable tool in exercise interventions is a computer-tailored website or app (where content material is adapted, with the aid of algorithms within the website or app, to the specific characteristics of a particular person). In 3 recent studies, Golsteijn et al [12], Trinh et al [13], and Kenfield et al [14] have all demonstrated the feasibility and acceptability of using web- or app-based tools to increase physical activity levels in individuals with prostate cancer (only Trinh et al [13] had individuals with metastatic cancer, 36%). However, these interventions focused on improving behaviors such as reducing sedentary levels and increasing moderate-to-vigorous physical activity levels. Furthermore, the three interventions did not provide tailored exercise programming [12-14]. Given that individuals with metastatic prostate cancer have varying levels of capacity and those with bone metastasis require tailored exercise programs that consider the location, extent, and type of metastatic lesion, personalized multimodal programs are exceptionally vital [5,6,8,15].

Engagement with digital physical activity interventions is considered important for their effectiveness, and thus evaluating the factors that influence engagement within tools such as *ExerciseGuide* is vital [16]. Perski et al [16] proposed a conceptual framework in which engagement with an intervention is influenced by factors such as the content and delivery of the tool, as well as the target population and environment. Delivery can be assessed by evaluating usability and the ease with which a platform can be used to attain a particular goal [17]. Acceptability is another concept that can be used to predict user engagement [18]. Acceptability is defined as “a multi-faceted construct that reflects the extent to which people delivering or receiving a healthcare intervention consider it to be appropriate, based on anticipated or experienced cognitive and emotional responses to the intervention” [19]. Therefore, following a user-centered approach, it is important to have the usability and acceptability of the intervention’s design and content assessed by individuals with metastatic prostate cancer.

Furthermore, the safety implications of computer-tailored exercise prescription in this population are unknown. It is necessary to determine whether individuals with metastatic prostate cancer can adequately replicate exercise without hands-on technique modification when needed. To answer these questions, we designed a laboratory-based study incorporating both quantitative and qualitative usability and acceptability user evaluations, as well as objective movement screening. This allows small-scale assessment of the intervention and iterative refinement before progressing to a larger-scale study [20].

### Aims

This study aims to (1) examine and refine the acceptability and usability of a web-based exercise intervention (known as *ExerciseGuide*) for individuals with metastatic prostate cancer and (2) examine the safety of video-guided resistance exercises used within the *ExerciseGuide* intervention.

## Methods

### Study Design

#### Overview

This study is a laboratory-based assessment that used both qualitative and quantitative approaches. This trial was registered in the Australian New Zealand Clinical Trials Registry (ACTRN12618001978257) and approved by the University of Adelaide Human Research Ethics Committee. Study materials, including the participant information sheet and data request forms, are available through the Open Science Framework.

#### *ExerciseGuide* Intervention Development

The design and development process of the web-based exercise website (*ExerciseGuide*) used a multidisciplinary approach (exercise physiology, behavioral science, public health, medical oncology, and urology) that was guided by the intervention mapping protocol [21] and preliminary research [6,22,23].

#### Participants and Screening

Men with metastatic prostate cancer were recruited using convenience sampling methods, which involved advertising the study through social media and intermediaries (oncologists, nurses, participant registries, and support groups). Previous evidence has shown that more than 80% of the usability issues can be detected with 5-9 participants and 90%-95% using 10-12 participants [24]; therefore, a sample size of approximately 10 participants was proposed.

**Figure 1:**
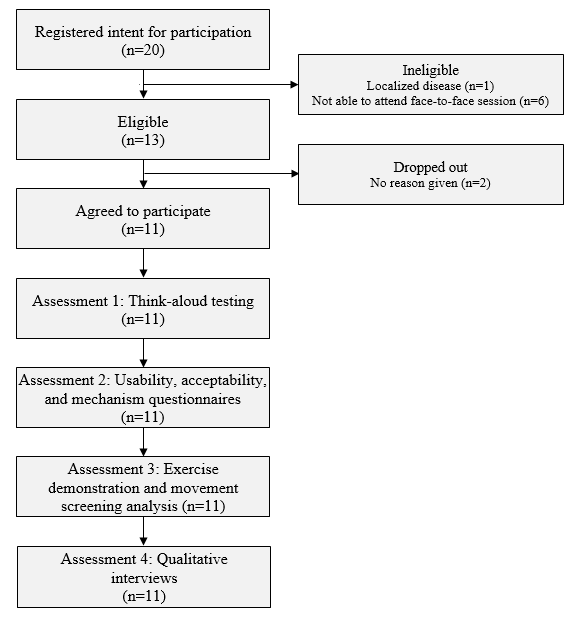
Participant flow chart for individuals with metastatic prostate cancer.

To be eligible, participants needed to be diagnosed with metastatic prostate cancer, able to obtain consent to participate from their physician, able to attend a single 90- to 120-minute face-to-face session at the University of Adelaide (Adelaide) or the University of Melbourne (Melbourne), confident of their ability to participate in some form of moderate resistance exercise for 5 minutes or more, and able to read and write in English. The participant flow is presented in Figure 1.

### Study Procedure

#### Overview

To investigate the study aims, four assessment blocks were used: (1) a think-aloud usability test, (2) questionnaires to assess usability and acceptability, (3) exercise demonstration and movement screening to determine the safety and potential efficacy of video-guided resistance exercises, and (4) qualitative interviews further assessing acceptability and perceived usefulness. In all, two iterative cycles were conducted, with website alterations made after the fifth and eleventh participants based on usability issues identified across the assessment blocks.

The participants were sent a link to the self-administered baseline questionnaire through REDCap (Research Electronic Data Capture; Vanderbilt University) 24 hours before arriving at the laboratory for testing. The questionnaire was used to collect general and prostate cancer–specific demographic data, including prostate-specific antigen score (ng/mL), time since disease diagnosis (years), and the number of bone metastases. Physical activity behavior was measured using the modified Godin Leisure-Time Exercise Questionnaire. The weekly frequencies (longer than 15 minutes) of vigorous, moderate, and light physical activities were weighted and summed to obtain a total score in units [25]. The 2-week test-retest reliability was found to be high [26]. The 12-item Short Form Survey, which is a reliable and valid instrument for adults with cancer, was used to quantify health-related quality of life [27]. Internet use was gauged based on a question used in the study by Short et al [28], and internet confidence (3 items rated on a 0-100 scale) questions were study specific.

#### Assessment Block 1: Usability Testing Using the Think-Aloud Testing Methodology

**Figure 2:**
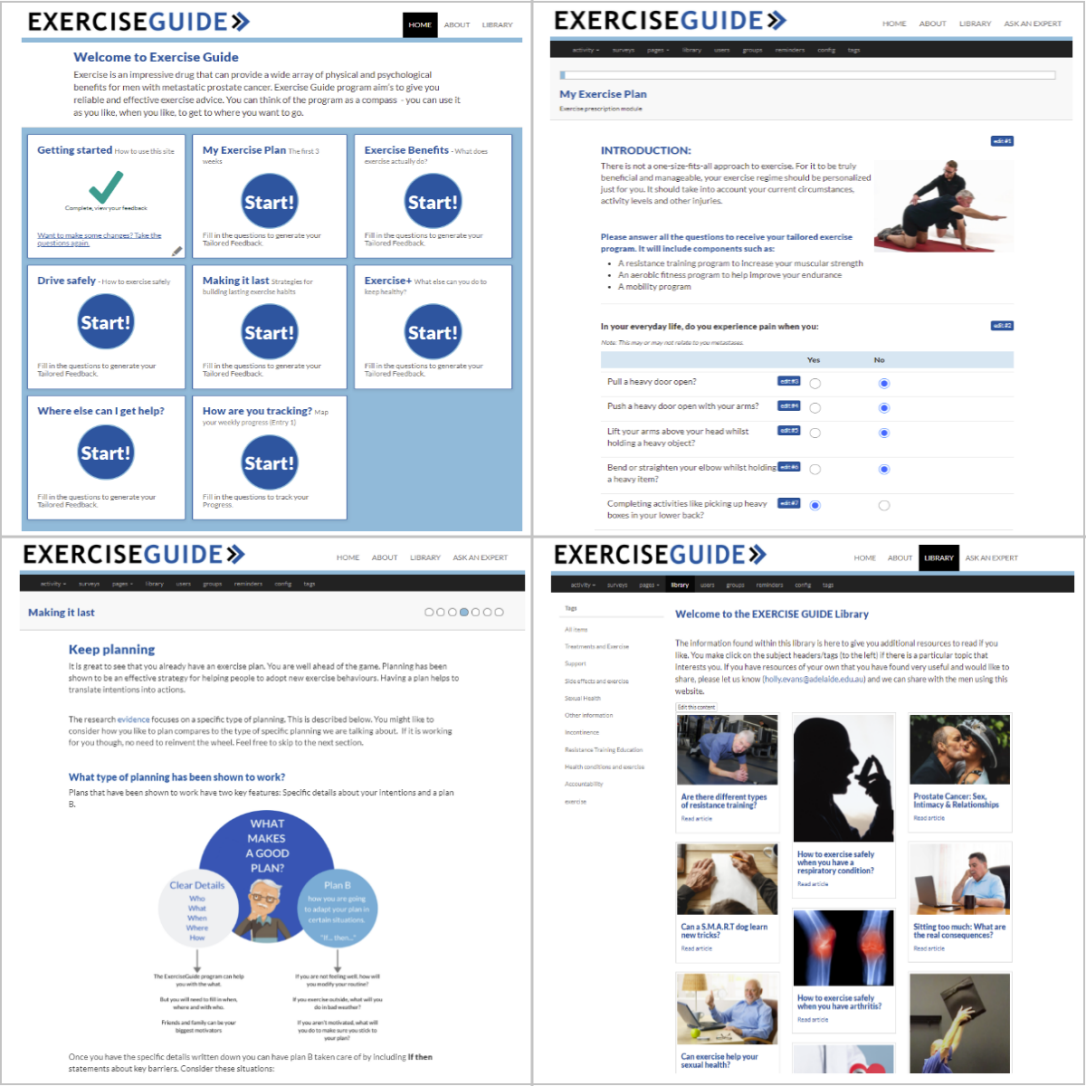
*ExerciseGuide* website screenshots of (1) the home page (top left), (2) Making It Last module tailoring questions (top right), (3) My Exercise Plan module (bottom left), and (4) library page (bottom right).

A concurrent think-aloud approach was used to identify usability issues within the website. This approach has been recognized as one of the most effective and commonly used tools to understand usability in eHealth work, especially when used in conjunction with other methods [17,29]. The laboratory location was chosen because laboratory studies have shown results similar to those obtained in field testing, while being time and resource efficient [30]. The *ExerciseGuide* website was presented on either a Windows (Microsoft Corporation) or Apple (Apple Inc) laptop, as chosen by the participants. A researcher (HELE) asked the participants to verbally narrate their thought processes and feelings while completing the designated tasks on the *ExerciseGuide* website (Multimedia Appendix 1). The tasks included logging in, answering module questions (to read tailored content), generating their personalized exercise prescription, watching videos, and identifying key tools such as the library and frequently asked questions (Figure 2). When the participants fell silent for approximately 30 seconds or became stuck in a particular task, they were encouraged to express what they were thinking. The think-aloud sessions were audiotaped, and written notes were taken by the researcher.

#### Assessment Block 2: Usability and Acceptability Questionnaires

A questionnaire was administered in private after the completion of think-aloud testing. Website usability was assessed using the System Usability Scale (SUS), which includes 10 questions rated from 1 (strongly disagree) to 5 (strongly agree) [31]. It is the most commonly used questionnaire for the assessment of perceived usability [32]. The reliability of the SUS (coefficient α) was high, and the concurrent validity was significantly correlated [32].

For the purpose of this study, 6 questions were used to determine participant perception of intervention acceptability using a 5-point Likert scale (from 1=strongly disagree to 5=strongly agree) [19,28]. The questions were used previously by Short et al [28], and the internal consistency of the SUS was found to be high [28]. The purpose was to examine if the website was interesting, credible, easy to understand, relevant, and if the participants were likely to recommend the website to a friend.

#### Assessment Block 3: Resistance Exercise Demonstration and Movement Screening Analysis

A qualified exercise physiologist (HELE) reviewed the resistance exercise prescription that the participants generated using the *ExerciseGuide* website within the think-aloud protocol to determine if any of the recommended exercises were inappropriate. Any exercise deemed unsafe based on the location of the metastases would not be completed. The participants were asked to replicate each exercise under the direct observation of the exercise professional. For each exercise, they were able to watch the exercise demonstration video and read the written instructions as many times as needed. The participants selected the resistance exercise band that they felt would produce an effort of 6-7 out of 10 on the OMNI Perceived Exertion Scale for Resistance Exercise and completed 8 repetitions. The participant was recorded using 2 iPads (Apple Inc; 30 frames per second, 1080p) mounted on tripods positioned orthogonal to each other. Camera 1 was positioned to record the sagittal movement plane and camera 2 the frontal plane [33]. The participants reported a verbal pain score (0-10) during and after the exercise and a verbal rating of perceived exertion (0-10) after the exercise. The exercise was halted if the pain level score was higher than 3 out of 10 or if the technique was unsafe.

The movement screening analysis was completed by 5 independent exercise physiologists, accredited by Exercise and Sports Science Australia, each with more than 5 years of clinical experience (Table 1). The video recordings of each resistance exercise were assessed using a standardized form developed by an exercise physiologist (HELE) a priori based on evidence-based movement quality assessment (Multimedia Appendix 2). Each exercise was individually scored in terms of both safety and efficacy items (between 6 and 8 items per exercise) on a scale of –1 (unsatisfactory, with major concerns) to 2 (good). The exercise physiologists were encouraged to provide notes regarding the movement issues where applicable. Before analysis, the scores were transformed to reflect a positive score ranging from 1 to 4 for each item. The item scores were then added to create an overall movement score. The information collected was also used to determine the interrater reliability of the tool among the experts.

**Table 1:** Reviewer (exercise physiologist) characteristics.

Reviewer	Occupational setting	Experience (years)	Gender	Current location
1	Private practice	9	Female	Victoria, Australia
2	Public health	9	Female	Victoria, Australia
3	University and private practice	20	Female	Queensland, Australia
4	Private practice	5	Female	New South Wales, Australia
5	Private practice	7	Male	South Australia, Australia

#### Assessment Block 4: Qualitative Interviews

Finally, the participants completed a one-on-one short semistructured interview with a researcher (HELE) to identify further technical issues, investigate user experiences, and obtain feedback to improve site content and usability. The interview guide consisted of 8 open-ended questions (Multimedia Appendix 3). The interviews were audiotaped and transcribed verbatim.

### Data Analysis

Quantitative analyses were performed using Jamovi software (version 1.6.3; The Jamovi project). Descriptive statistics were calculated with mean values and SDs for normally distributed data and medians with range or percentage for nonnormal and categorical data. In addition, intraclass correlation coefficients were calculated to determine the interrater reliability of the overall exercise movement screening scores. 

The qualitative data collected were analyzed using thematic analysis as described by Braun and Clarke [34]. This process has previously been used to analyze data from usability think-aloud studies and involves data familiarization, generation of initial codes, theme identification, refining of themes, and theme names [34]. In this study, an initial set of themes was produced and organized by the first author (HELE) using Microsoft Excel (Microsoft Corporation) and iteratively refined with a second author (CES), leading to the discovery of new themes or renaming of existing themes. Descriptive quotes illustrating the themes were identified and reviewed by all the authors. The results were reported based on the topic area (ie, usability, acceptability, and safety) rather than through assessment block to aid interpretation in accordance with the study aims.

### Ethics Approval and Consent to Participate

This study was performed in accordance with the principles of the Declaration of Helsinki. Ethical clearance was obtained from the University of Adelaide Research Ethics Committee (H-2017-174). The participants were required to provide signed, freely given informed consent at the time of enrollment.

## Results

### Participant Characteristics

A total of 11 men with metastatic prostate cancer were recruited for this study, and their characteristics are presented in Table 2. Most of the participants were married and residing in a major city. There were no significant differences in the characteristics of the participants between cycle 1 and cycle 2. Confidence in internet use was moderate on average.

**Table 2:** Participant characteristics (N=11).

Characteristics		Cycle 1 (n=5)	Cycle 2 (n=6)	Total (N=11)
Age (years), mean (SD)		74.8 (7.2)	72 (6.5)	73.37 (6.7)
BMI (kg/m^2^), mean (SD)		27.13 (2.2)	29.9 (6.1)	28.6 (4.7)
Married, n (%)		4 (80)	6 (100)	10 (91)
**Location, n (%)**				
	Major city	4 (80)	6 (100)	10 (91)
	Very remote	1 (20)	0 (0)	1 (9)
**Education, n (%)**				
	Secondary school	3 (60)	1 (17)	4 (36)
	Trade, technical certificate, or diploma	2 (40)	1 (17)	3 (27)
	University or other tertiary	0 (0)	2 (33)	2 (18)
	Postgraduate	0 (0)	2 (33)	2 (18)
**Employment, n (%)**				
	Employed full time	1 (20)	0 (0)	1 (9)
	Employed part time	0 (0)	0 (0)	0 (0)
	Self-employed	0 (0)	1 (16.7)	1 (9)
	Retired	3 (60)	4 (67)	7 (64)
	Volunteer	1 (20)	1 (17)	2 (18)
Current PSA^a^ level, ng/mL, median (IQR)		0.32 (0-6.32)	0.015 (0.10-2.23)	0.02 (0-4.17)
Time since metastatic disease diagnosis, years, mean (SD)		2 (0.8)	2.6 (3.1)	2.3 (2.2)
Individuals with ≥1 bone lesion, n (%)		4 (80)	5 (83)	9 (82)
Comorbidities, mean (SD)^b^		2.8 (1.5)	2.7 (0.9)	2.7 (1.2)
**Self-reported quality of life (SF-12^c^), mean (SD)^d^**				
	PCS-12^e^ (physical score)	46.23 (5.6)	36.80 (12.8)	41.09 (10.9)
	MCS-12^f^ (mental score)	58.9 (3.3)	52.1 (4.3)	55.3 (5)
**Self-reported physical activity, mean (SD)**				
	Aerobic physical activity (GLTEQ^g^ units)^h^	53.8 (22.3)	32.8 (21.9)	42.4 (23.7)
	Resistance training sessions (per week)	2.2 (1.7)	2.2 (1.3)	2.2 (1.5)
**Average internet use (hours per week), n (%)**				
	≥6	1 (20)	4 (67)	5 (45)
	3-5	2 (40)	0 (0)	3 (18)
	2-3	1 (20)	1 (17)	2 (18)
	≥1	0 (0)	1 (17)	1 (9)
	None	1 (20)	0 (0)	1 (9)
**Confidence to use the internet (0-100 scale)^i^, mean (SD)**				
	Finding information on the internet	63.6 (30.1)	72.2 (36.1)	68.3 (33.7)
	Using the internet to interact with others (eg, social media)	53.2 (36.4)	55.2 (20)	54.3 (28.6)
	Using an interactive website to help increase physical activity	44 (39.9)	46.3 (40.9)	45.3 (40.5)

^a^PSA: prostate-specific antigen.

^b^Comorbidities include hypertension, osteoarthritis, chronic nonspecific back pain, osteoporosis, type 2 diabetes, cardiovascular disease, and mental health conditions.

^c^SF-12: 12-item Short Form Survey.

^d^Scores range from 0 to 100, where 0 implies the lowest level of quality of life, and 100 indicates the highest level of quality of life.

^e^PCS-12: Physical Component Score.

^f^MCS-12: Mental Component Score.

^g^GLTEQ: Godin Leisure-Time Exercise Questionnaire.

^h^Self-reported physical activity level from the Godin Leisure-Time Exercise Questionnaire. Physical activity score (units) = strenuous (9 METs × times/week) + moderate (5 METs × times/week) + light (3 METs × times/week). One metabolic equivalent (MET) is the amount of oxygen consumed while sitting at rest and is equal to 3.5 mL of oxygen per kg body weight × minutes [26].

^i^Confidence in using the internet scored on a scale from 0 to 100 (0=not confident at all, 100=extremely confident).

### Usability

#### Overview

There was an increase in the usability score from 60 (SD 2.9) to 69.6 (SD 2.2) out of 100 between cycle 1 and cycle 2, indicating that the changes suggested by the participants increased the usability to *slightly above average* (based on industry standards) [31]. Qualitative feedback regarding usability is summarized below. A list of changes made to the website based on user feedback is presented in Multimedia Appendix 4.

**Figure 3:**
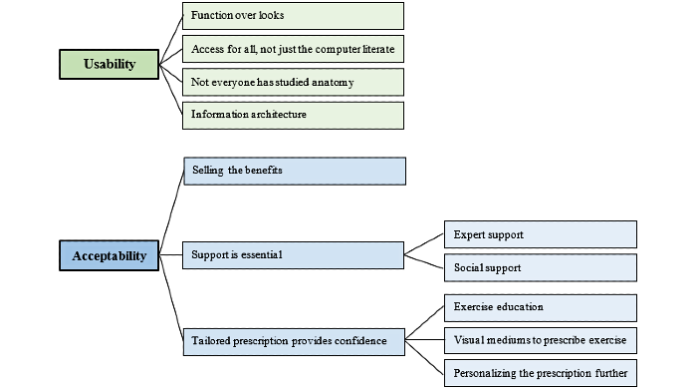
Coding structure derived from thematic analysis.

The qualitative usability feedback centered around four themes, as shown in Figure 3. The participants discussed the need for simplicity in the website design and suggested that the function should trump looks, that the design needs to account for those with lower computer literacy, and that the terminology should be simplified but not come across as patronizing. They also observed that efforts to reduce information overload are required.

#### Theme 1: Function Over Looks

The participants (5/11, 45%) reported that esthetics were not as important as the functionality of a website:“ It is not very ornate, but I think the simplicity is helpful because it gives you the specifics, and it’s not offensive in any way.” [ID 03, aged 78 years, <1 year after diagnosis]. Of the 11 participants, 2 (18%) reported that this desire was linked to their gender:

I didn’t need it to look more pretty, I don’t care about that...a lot of males in my age group wouldn’t be all that worried about that either.

ID 07, aged 72 years, 3 years after diagnosis

In general, the participants liked that the website was plain but straightforward, and that made the website user friendly.

#### Theme 2: Access for All, Not Just the Computer Literate

Of the 11 participants, 3 (27%) believed that aspects of the website were not designed for individuals with lower literacy levels:

You are 80% simple, but I still looked at it and went ehhhh...it was a bit daunting.

ID 11, aged 65 years, 1 year after diagnosis

Questionnaires used to tailor content and the website navigation videos should be further simplified. Of the 11 participants, 3 (27%) could not get the videos to play, and 5 (45%) found that the introduction videos moved through information too quickly. Of the 11 participants, 1 (9%) man with low computer literacy could not complete the think-aloud protocol without support and preferred an option where information could be printed for him:

I’m very unfamiliar with them [computers]. If you wrote it all on a piece of paper, then it would be easy, but it’s not like that.

ID 02, aged 82 years, 4 years after diagnosis

In addition, another participant suggested that the use of closed captions would increase usability for individuals with hearing concerns.

#### Theme 3: Not Everyone Has Studied Anatomy

The participants also desired more lay language in the health education provided. The use of medical terminology hampered usability in this population: “The explanations need to be for someone like me who hasn’t done anatomy.” [ID 06, aged 73 years, 1 year after diagnosis]. Of the 11 participants, 5 (45%) men questioned words such as androgen deprivation therapy, neutrophils, and hypertrophy. Information should be presented in laymen’s language without being patronizing. Of the 11 participants, 1 (9%) suggested that terminology is still useful but could be linked to a quick and easy definition: “Where we have terminology, put in there so if the person hovers their mouse or their stylus over the word, then the definition would pop up?” [ID 07, aged 72 years, 3 years after diagnosis].

#### Theme 4: Information Architecture

The flexible modular design was seen as clear and user friendly by 55% (6/11) of the participants. The modules reduced the content into smaller bite-sized chunks and allowed simple navigation: “I like the way it is modulised, so I can come into it any time and examine any part of it, then go away and come back and do another module later.” [ID 09, aged 78 years, 6 years after diagnosis]. Most of the men (8/11, 73%) appreciated the flexible nature, where they could read the information that was most meaningful to them.

Furthermore, the use of computer tailoring was a standout for many of the participants (5/11, 45%) because it reduced the amount of content within the website:

I thought the way it was designed to cater for individual people instead of a one-size-fits-all...That was a standout I thought.

ID 01, aged 74 years, 8 years after diagnosis

However, of the 11 participants, 4 (36%) still felt that the website was very content dense and that modules and associated tailoring questions could be condensed or split. Furthermore, of the 11 participants, 1 (9%) believed that introducing the website and providing examples of how the website can be used may improve usability:

Introducing the options of how to use the website at the outset, either sequentially or dipping in where appropriate. Going through the whole thing end to end, that’s fairly daunting because of the amount of information.

ID 08, aged 64 years, 2 years after diagnosis

### Acceptability

#### Overview

Overall, the participants’ perceptions of the website were largely positive across both cycles (Table 3). Of note, the participants were in strong agreement that they would be happy to recommend the website to a friend with the same diagnosis (11/11, 100% reporting agree or strongly agree). The lowest score revolved around the ease of understanding of the information presented. A list of changes made to the *ExerciseGuide* intervention based on user feedback is presented in Multimedia Appendix 5 [35].

Each item was scored on a 5-point Likert scale ranging from 1 (strongly disagree) to 5 (strongly agree). The overall acceptability score was the sum of the scores from all 6 questions. The total overall acceptability mean score for cycle 1 was 24.2 (SD 1.1) and cycle 2 was 26.3 (SD 2.1). The combined mean score was 25.4 (SD 2).

The participants’ qualitative feedback centered around the factors that they believed would improve the website (Figure 3). More strongly, selling the benefits of exercise was deemed important, as was support from both experts and those close to the participants. Finally, confidence in completing the exercises safely and effectively was also noted.

**Table 3:** Website acceptability ratings (N=11).

Acceptability item	Cycle 1 (n=5), mean (SD)	Cycle 2 (n=6), mean (SD)	Total (N=11), mean (SD)
The information provided to me on the website was interesting.	4 (0)	4.33 (0.5)	4.2 (0.4)
The information provided to me on the website was credible.	4 (0)	4.5 (0.5)	4.2 (0.5)
The information provided to me on the website was easy to understand.	3.6 (0.9)	4.2 (0.4)	3.9 (0.7)
The information provided to the website was relevant to me personally.	4 (0)	4.33 (0.52)	4.2 (0.4)
I would recommend the website to a friend with the same diagnosis as me.	4.4 (0.6)	4.7 (0.5)	4.6 (0.5)
The website seems like it was written for someone like me in mind.	4.2 (0.8)	4.3 (0.5)	4.3 (0.7)

#### Theme 1: Selling the Benefits

Of the 11 participants, 3 (27%) noted the importance of exercise, and 2 (18%) believed that there was not enough emphasis on explaining the benefits of exercise:

You need to sell the story. Explain the research behind it, that it’s not a myth. That there is lots of evidence with prostate cancer, that Australia is leading the field.

ID 08, aged 64 years, 2 years after diagnosis

Another participant believed that the website should sell the benefits of exercise as soon as possible, rather than just addressing the benefits in one module that may not be accessed:

The home page doesn’t explain enough...you are trying to sell an idea to a person who is going to say fuey, I don’t need that...you are selling the concept.

ID 04, aged 63 years, <1 year after diagnosis

#### Theme 2: Support Is Essential

Support by experts, family, and friends emerged as an important aspect of the intervention to improve adherence to an exercise program and help guide the website’s use.

##### Expert Support

Expert support was highlighted as a method of support deemed valuable by 36% (4/11) of the participants. Having access to an expert may increase confidence in the exercises prescribed because the participants could ask questions about the website, have exercises modified, identify exercise barriers and facilitators, and receive external motivation:

It would be good to have a backup, some actually contacting the person saying how’s it going, did you like the exercises? You know...just to be a buddy.

ID 07, aged 72 years, 3 years after diagnosis

The desired regularity of contact varied between weekly and monthly interactions, and video conferencing, phone calls, and emails were all acceptable. Of the 11 participants, 2 (18%) noted that the support would only be useful if it were personalized rather than automated.

##### Social Support

A supportive social environment was reported as the other possible facilitator to intervention adherence: “The real attraction about going out [to exercise with friends] is to stop midway through for a coffee and a chat, and I think that makes a big thing*.*” [ID 01, aged 74 years, 8 years after diagnosis]. Of the 11 participants, 2 (18%) believed that encouraging participants to develop, reconnect, or enhance social support structures such as family or friends to prompt and support exercise adherence would be effective.

#### Theme 3: Tailored Prescription Provides Confidence

The participants discussed a lack of confidence in exercising because they were unsure of what exercises were safe and effective. Supplying tailored prostate cancer–specific exercise information, which could be modified to suit the participant, was highlighted as a way to increase confidence.

##### Exercise Education

There was an appreciation that the website provided tailored prostate cancer–specific information: “I understand that it is good to exercise, but I haven’t had a definition of how much to do, and this may give me that information, which will be good.” [ID 03, 78 years old, newly diagnosed]. In general, the multimodal exercise program was positively received by all participants: “They [the exercises] were within my abilities but there again, with the different therabands, it’s probably going to be suitable for a big range of people.” [ID 01, aged 74 years, 8 years after diagnosis]*.* Of the 11 participants, 3 (27%) wanted additional options of aerobic activity, rather than just walking or cycling, and 2 (18%) requested a tailored stretching program.

##### Visual Mediums to Prescribe Exercise

Video-based exercise prescription was seen as an appropriate and useful medium by all participants. In general, the participants typically used the on-demand videos rather than the written instructions:

The videos were great. The presenter was well spoken, you could hear what he was saying. They were crisp and clear. Easy to follow. Easy to backtrack.

ID 04, aged 63 years, newly diagnosed

Of the 11 participants, 9 (82%) reported feeling confident in completing the exercises without additional support after watching the videos, and 4 (36%) were comfortable returning to the videos as often as needed to ensure that their technique was correct. Of the 11 participants, 1 (9%) noted that the exercise trainer could have more readily explained what muscles should be focused on and explain why the exercise would be useful from a functional perspective:

The trainer could have explained what muscles he was using. That way, the person knows why he is doing that exercise; they are not just a sheep following a thing...He did on some, but he needed to acknowledge why.

ID 07, aged 72 years, 3 years after diagnosis

There was a perception that many men may overload themselves when exercising, which may lead to an increased risk of injury (2/11, 18%). Providing simple ways to monitor their exercise intensity was highlighted and may reduce the risk of injury in this population:

I think that this [rate of perceived exertion information] is really important. Sweeping generalization comes up, but men tend to push themselves slightly harder than they should. They are competing with themselves, and that can lead to injury.

ID 06, aged 73 years, 1 year after diagnosis

##### Personalizing the Prescription Further

Multiple participants (7/11, 64%) provided further information to support individual autoregulation. Of the 11 participants, 4 (36%) discussed techniques to increase or decrease their exercise intensity to suit how they feel on the day, and 1 (9%) noted that not all participants wanted to make progress regarding their exercise intensity. Maintenance of strength and aerobic fitness are noteworthy goals, especially for those who do not enjoy exercise. Tailoring messages to avoid pushing individuals into making progress regarding their exercise intensity may improve adherence:

Once you get to a fitness level that suits you, why push it. Where here is it’s saying you need to make it harder to challenge yourself...I don’t think we need to challenge ourselves. I think it is just a challenge just to exercise for some people.

ID 04, aged 63 years, <1 year after diagnosis

Finally, 27% (3/11) of the men found that the program needed to include modifications to suit those already doing some form of exercise to reduce confusion and possible overload. As long as safety concerns have been addressed, the *ExerciseGuide* program should sit within an individual’s exercise schedule, rather than completely changing it.

#### Safety-Movement Screening

The website prescribed 6.6 (SD 1.5) exercises per participant on average. A total of 18 of the possible 25 exercises available were prescribed. No exercises were removed for safety reasons, as judged by the participant or by a supervising exercise physiologist. The participants reported a mean rate of perceived exertion score of 6.2 (SD 1.2) and a mean verbally reported pain score of 0.2 (SD 0.3) (possible range 0-10). Of the 11 participants, 2 (18%) reported a pain level of 3-4 out of 10 on 3 different exercises (single leg lift, seated knee extension, and seated march). On both accounts, the pain was linked to previous knee injuries and was not recorded as bone pain. Pain resolved once the movement ceased.

Overall, no exercises were deemed unsatisfactory, with all meeting the cutoff point for safety defined as a rating of *satisfactory* or *good,* as demonstrated in Multimedia Appendix 6. Only reviewer 2 scored 1 exercise as unsatisfactory (seated triceps extension). However, it is noteworthy that the intraclass correlation coefficients for the combined item scores demonstrated very low interrater reliability among the assessors (0-0.592).

When viewing the mean scores of the individual items within each exercise, it was clear that overall, participants set up satisfactorily (3.6, SD 0.3 out of 4). Of the 11 participants, only 2 (18%) set up in an unsatisfactory manner: 1 in the seated row and 1 in the incline push-up. On average, the participants could complete the movements in a slow, controlled manner (3.8, SD 0.2 out of 4) as directed. However, it was notable in the triceps extension and bicep curl exercises that the individuals did not satisfactorily maintain appropriate elbow positions that would isolate the target muscle groups, increasing loads around the thoracic region. In addition, in the lower body exercises that required resisted knee flexion and extension, the individuals did not satisfactorily maintain their torso vertical, which may lead to additional strain through the anterior hip and lumbar spine.

## Discussion

### Principal Findings

This is the first study to examine the acceptability, usability, and safety aspects of a web-based exercise intervention tailored directly for individuals with metastatic prostate cancer. Overall, the participants found the tailored intervention acceptable and a user-friendly method of delivering credible health-based education, exercise prescription, and behavioral change advice. This is in line with previous studies in older adults with localized prostate cancer [14].

The participants were more interested in functionality than esthetics. This is in accordance with the Technology Acceptance Model, which posits that use is determined by the perceived ease of use and usefulness of technology [36]. Alterations made after the first cycle, including increased text size (from 12 point to 15 point), greater format consistency, and education to upskill users in website use, mirror existing eHealth recommendations [37].

The use of computer tailoring within the *ExerciseGuide* intervention was viewed as a strength by the participants. Older adults have been reported to have difficulty filtering out useful information from generalized text because of changes in working memory [38]. Tailoring information ensures personal relevance, individualized exercise prescription, and limitation of superfluous information [39]. Notably, additional tailoring occurred after the first iterative cycle, with the aim of increasing the personalization of exercise and reducing the amount of content. An improvement in both relevance and ease of understanding the scores was achieved in cycle 2. However, the use of questionnaires within each module to collate tailoring information still has some limitations. Ghalibaf et al [40] reported decreased usability and acceptability because participants find providing the system with information time consuming. Further research is needed to determine other user-friendly and accurate methods of information collection.

There was disagreement among the participants regarding the use of medical terminology within the intervention. Previous studies corroborate the viewpoint of several of the participants who deemed simplified language to be important for usability [41,42]. However, other participants in this study appreciated the use of medical descriptions. As such, if medical terminology is used, it should be clearly explained, thus providing a chance to improve the health literacy of participants.

Most of the participants emphasized the need for multiple avenues of personalized expert support throughout the *ExerciseGuide* intervention to ensure higher levels of uptake, adherence, and safety. Haberlin et al [43] reported a need for on-site exercise prescription and behavioral change support at the start of a physical activity eHealth intervention. However, the participants in this study were comfortable with remote telehealth technology such as teleconferencing (otherwise known as real-time video counseling), phone conferencing, email, and instant messaging as vehicles of support from health professionals. It is theorized that the injection of this type of technology into home-based exercise prescription can increase supervision and improve the participant–health professional relationship while still being a cost-effective and accessible intervention [44,45]. Interestingly, Byaruhanga et al [46] reported that real-time video counseling could enhance physical activity behaviors in clinical populations compared with usual care. However, other telehealth tools (eg, email and SMS) also have benefits such as accessibility, satisfaction, and comfort [47]. Further research is still needed to explore the efficacy of different types of technology for exercise prescription and support in this population and others.

The computer-tailored resistance exercise prescription was effective at prescribing clinically recommended exercises to the patients in this study. The participants reported finding the resistance exercise demonstration videos easy to follow and could replicate them to at least a satisfactory level, as judged by the novel movement screen. However, the movement screen analysis indicated that when prescribing distance-based exercise programs to individuals with metastatic prostate cancer, exercise professionals should focus on body positioning to allow greater isolation of the targeted muscles and reduce the mechanical load on bone lesions. Highlighting proper positioning by emphasizing the important cues in the video, explaining why isolation is important, and encouraging visual cues (ie, mirrors) are all methods that could be beneficial.

### Strengths and Limitations

A strength of this evaluation was the emphasis on user-centered assessment and the novel approach to appraising exercise prescription safety within a tailored web-based intervention. However, this study should be evaluated within its limitations. Overall, the sample population consisted of Caucasian, English-speaking men with a relatively high level of exercise activity and internet experience and may not reflect the full range of user experiences. Second, the methodology did not include safety testing for aerobic exercise because of resource constraints, and the interclass correlation for the movement screening tool was very low. Third, the study recruited a small number of participants. The sample size is typical for usability testing, and the researchers felt that data saturation for the qualitative components was achieved. However, it is possible that a greater range of feedback would have been captured in a larger sample. Finally, the sample website did not contain all the behavioral change and other educational content planned for the full website. The authors felt that the participants would experience the main components of the abridged website’s design and content.

### Conclusions

This preliminary study exemplifies how evidence-based theory and the target users’ input can facilitate the development of a web-based exercise intervention to meet the needs and preferences of this population. On account of the iterative nature of this study, numerous issues were identified and resolved. A prominent finding was the request for distance-based personalized support as an addition to the intervention in the form of video conferencing, phone conferencing, or SMS. Overall, the design and content within *ExerciseGuide* were viewed as acceptable and user friendly. The resistance training algorithms were shown to provide appropriate content safely, and users could replicate the exercise technique unaided to a satisfactory level. This study will be used to further refine the *ExerciseGuide* website. The next phase of testing will be conducted to determine the feasibility and preliminary efficacy of the tool [35].

## Appendix

Multimedia Appendix 1 Think-aloud test instructions.

Multimedia Appendix 2 Movement screening proforma.

Multimedia Appendix 3 Semistructured interview guide.

Multimedia Appendix 4 Think-aloud modifications.

Multimedia Appendix 5 <italic>ExerciseGuide</italic> intervention-specific changes pre- and postusability study.

Multimedia Appendix 6 Movement screening scores and intraclass correlation to estimate interrater reliability.

## Abbreviations

REDCap: Research Electronic Data Capture
SUS: System Usability Scale

## References

[1] (2021). Cancer Data in Australia. Australian Institute of Health and Welfare.

[2] (2017). Prostate Cancer in Australia. Australian Institute of Health and Welfare.

[3] Litwin MS, Tan H (2017). The diagnosis and treatment of prostate cancer: a review. J Am Med Assoc.

[4] Chambers SK, Hyde MK, Laurie K, Legg M, Frydenberg M, Davis ID, Lowe A, Dunn J (2018). Experiences of Australian men diagnosed with advanced prostate cancer: a qualitative study. BMJ Open.

[5] Hart NH, Galvão DA, Newton RU (2017). Exercise medicine for advanced prostate cancer. Curr Opin Support Palliat Care.

[6] Galvão DA, Taaffe DR, Spry N, Cormie P, Joseph D, Chambers SK, Chee R, Peddle-McIntyre CJ, Hart NH, Baumann FT, Denham J, Baker M, Newton RU (2018). Exercise preserves physical function in prostate cancer patients with bone metastases. Med Sci Sports Exerc.

[7] Cormie P, Newton RU, Spry N, Joseph D, Taaffe DR, Galvão DA (2013). Safety and efficacy of resistance exercise in prostate cancer patients with bone metastases. Prostate Cancer Prostatic Dis.

[8] Hayes SC, Newton RU, Spence RR, Galvão DA (2019). The exercise and sports science Australia position statement: exercise medicine in cancer management. J Sci Med Sport.

[9] Mina DS, Petrella A, Currie KL, Bietola K, Alibhai SM, Trachtenberg J, Ritvo P, Matthew AG (2015). Enablers and barriers in delivery of a cancer exercise program: the Canadian experience. Curr Oncol.

[10] Sheill G, Guinan E, Neill LO, Hevey D, Hussey J (2018). The views of patients with metastatic prostate cancer towards physical activity: a qualitative exploration. Support Care Cancer.

[11] Brown M, Murphy M, McDermott L, McAneney H, O'Sullivan JM, Jain S, Prue G (2019). Exercise for advanced prostate cancer: a multicomponent, feasibility, trial protocol for men with metastatic castrate-resistant prostate cancer (EXACT). Pilot Feasibility Stud.

[12] Golsteijn RH, Bolman C, Peels DA, Volders E, de Vries H, Lechner L (2017). A web-based and print-based computer-tailored physical activity intervention for prostate and colorectal cancer survivors: a comparison of user characteristics and intervention use. J Med Internet Res.

[13] Trinh L, Arbour-Nicitopoulos KP, Sabiston CM, Berry SR, Loblaw A, Alibhai SM, Jones JM, Faulkner GE (2018). RiseTx: testing the feasibility of a web application for reducing sedentary behavior among prostate cancer survivors receiving androgen deprivation therapy. Int J Behav Nutr Phys Act.

[14] Kenfield SA, van Blarigan EL, Ameli N, Lavaki E, Cedars B, Paciorek AT, Monroy C, Tantum LK, Newton RU, Signorell C, Suh JH, Zhang L, Cooperberg MR, Carroll PR, Chan JM (2019). Feasibility, acceptability, and behavioral outcomes from a technology-enhanced behavioral change intervention (prostate 8): a pilot randomized controlled trial in men with prostate cancer. Eur Urol.

[15] Newton RU, Kenfield SA, Hart NH, Chan JM, Courneya KS, Catto J, Finn SP, Greenwood R, Hughes DC, Mucci L, Plymate SR, Praet SF, Guinan EM, van Blarigan EL, Casey O, Buzza M, Gledhill S, Zhang L, Galvão DA, Ryan CJ, Saad F (2018). Intense exercise for survival among men with metastatic castrate-resistant prostate cancer (INTERVAL-GAP4): a multicentre, randomised, controlled phase III study protocol. BMJ Open.

[16] Perski O, Blandford A, West R, Michie S (2017). Conceptualising engagement with digital behaviour change interventions: a systematic review using principles from critical interpretive synthesis. Transl Behav Med.

[17] Broekhuis M, van Velsen L, Hermens H (2019). Assessing usability of eHealth technology: a comparison of usability benchmarking instruments. Int J Med Inform.

[18] Perski O, Short CE (2021). Acceptability of digital health interventions: embracing the complexity. Transl Behav Med.

[19] Sekhon M, Cartwright M, Francis JJ (2017). Acceptability of healthcare interventions: an overview of reviews and development of a theoretical framework. BMC Health Serv Res.

[20] Czajkowski SM, Powell LH, Adler N, Naar-King S, Reynolds KD, Hunter CM, Laraia B, Olster DH, Perna FM, Peterson JC, Epel E, Boyington JE, Charlson ME (2015). From ideas to efficacy: The ORBIT model for developing behavioral treatments for chronic diseases. Health Psychol.

[21] Bartholomew LK, Mullen PD (2011). Five roles for using theory and evidence in the design and testing of behavior change interventions. J Public Health Dent.

[22] Forbes CC, Finlay A, McIntosh M, Siddiquee S, Short CE (2019). A systematic review of the feasibility, acceptability, and efficacy of online supportive care interventions targeting men with a history of prostate cancer. J Cancer Surviv.

[23] Evans HE, Forbes CC, Vandelanotte C, Galvão DA, Newton RU, Wittert G, Chambers S, Kichenadasse G, Brook N, Girard D, Short CE (2021). Examining the priorities, needs and preferences of men with metastatic prostate cancer in designing a personalised ehealth exercise intervention. Int J Behav Med.

[24] Virzi RA (2016). Refining the test phase of usability evaluation: how many subjects is enough?. Hum Factors.

[25] Zopf EM, Newton RU, Taaffe DR, Spry N, Cormie P, Joseph D, Chambers SK, Baumann FT, Bloch W, Galvão DA (2017). Associations between aerobic exercise levels and physical and mental health outcomes in men with bone metastatic prostate cancer: a cross-sectional investigation. Eur J Cancer Care.

[26] Godin G, Shephard RJ (1985). A simple method to assess exercise behavior in the community. Can J Appl Sport Sci.

[27] Bhandari NR, Kathe N, Hayes C, Payakachat N (2018). Reliability and validity of SF-12v2 among adults with self-reported cancer. Res Soc Adm Pharm.

[28] Short CE, Rebar A, James EL, Duncan MJ, Courneya KS, Plotnikoff RC, Crutzen R, Vandelanotte C (2017). How do different delivery schedules of tailored web-based physical activity advice for breast cancer survivors influence intervention use and efficacy?. J Cancer Surviv.

[29] Jaspers MW, Steen T, van den Bos C, Geenen M (2004). The think aloud method: a guide to user interface design. Int J Med Inform.

[30] Kaikkonen A, Kekäläinen A, Cankar M, Kallio T, Kankainen A (2005). Usability testing of mobile applications: a comparison between laboratory and field testing. J Usability Stud.

[31] Brooke J (1996). SUS - a quick and dirty usability scale. Usability Eval Ind.

[32] Lewis JR (2018). The system usability scale: past, present, and future. Int J Human Computer Interact.

[33] Bennett H, Davison K, Arnold J, Martin M, Wood S, Norton K (2019). Reliability of a movement quality assessment tool to guide exercise prescription (MovementScreen). Int J Sports Phys Ther.

[34] Braun V, Clarke V (2006). Using thematic analysis in psychology. Qual Res Psychol.

[35] Evans HE, Forbes CC, Galvão DA, Vandelanotte C, Newton RU, Wittert G, Chambers S, Vincent AD, Kichenadasse G, Brook N, Girard D, Short CE (2021). Evaluating a web- and telephone-based personalised exercise intervention for individuals living with metastatic prostate cancer (ExerciseGuide): protocol for a pilot randomised controlled trial. Pilot Feasibility Stud.

[36] Gücin N, Berk ÖS (2015). Technology acceptance in health care: an integrative review of predictive factors and intervention programs. Soc Behav Sci.

[37] Berkowsky RW, Czaja SJ (2018). Challenges associated with online health information seeking among older adults. Aging Technol Heal.

[38] Bolle S, Romijn G, Smets EM, Loos EF, Kunneman M, van Weert JC (2016). Older cancer patients' user experiences with web-based health information tools: a think-aloud study. J Med Internet Res.

[39] Finlay A, Evans H, Vincent A, Wittert G, Vandelanotte C, Short CE (2020). Optimising web-based computer-tailored physical activity interventions for prostate cancer survivors: a randomised controlled trial examining the impact of website architecture on user engagement. Int J Environ Res Public Health.

[40] Ghalibaf AK, Nazari E, Gholian-Aval M, Tara M (2019). Comprehensive overview of computer-based health information tailoring: a systematic scoping review. BMJ Open.

[41] Børøsund E, Mirkovic J, Clark MM, Ehlers SL, Andrykowski MA, Bergland A, Westeng M, Nes LS (2018). A stress management app intervention for cancer survivors: design, development, and usability testing. JMIR Form Res.

[42] Osipenko L, Gajraj E, Thomas D (2019). NICE guidance and health technology assessment. Clinical Pharmacy Education, Practice and Research: Clinical Pharmacy, Drug Information, Pharmacovigilance, Pharmacoeconomics and Clinical Research.

[43] Haberlin C, O' Donnell DM, Moran J, Broderick J (2020). Perceptions of eHealth-enabled physical activity interventions among cancer survivors: mixed methods study. JMIR Cancer.

[44] Bland KA, Bigaran A, Campbell KL, Trevaskis M, Zopf EM (2020). Exercising in isolation? The role of telehealth in exercise oncology during the COVID-19 pandemic and beyond. Phys Ther.

[45] Gell NM, Grover KW, Savard L, Dittus K (2020). Outcomes of a text message, fitbit, and coaching intervention on physical activity maintenance among cancer survivors: a randomized control pilot trial. J Cancer Surviv.

[46] Byaruhanga J, Atorkey P, McLaughlin M, Brown A, Byrnes E, Paul C, Wiggers J, Tzelepis F (2020). Effectiveness of individual real-time video counseling on smoking, nutrition, alcohol, physical activity, and obesity health risks: systematic review. J Med Internet Res.

[47] Orlando JF, Beard M, Kumar S (2019). Systematic review of patient and caregivers' satisfaction with telehealth videoconferencing as a mode of service delivery in managing patients' health. PLoS One.

