# Supplementary material for: Usability, Acceptability, and Safety Analysis of a Computer-Tailored Web-Based Exercise Intervention (ExerciseGuide) for Individuals With Metastatic Prostate Cancer: Multi-Methods Laboratory-Based Study
Source: JMIR Cancer. 2021 Jul 28;7(3):e28370. doi: 10.2196/28370 (PMC8367181; doi:10.2196/28370)
Supplement: Multimedia Appendix 1 [file cancer_v7i3e28370_app1.docx]

**Multimedia Appendix 1:** **Think aloud test instructions**

Researcher:

During this part, I will be working from a script to ensure my instructions are the same for all participants. We will ask you to use the website to complete a set of tasks. As you do these tasks, I’m going to ask you as much as possible to try to think out loud: to say what you’re looking at, what you’re trying to do, and what you’re thinking.

It’s really important to know that we are only testing the site, not you. You can’t do or say anything wrong here. Let us know at any time if there’s something you like, dislike, is confusing etc. I promise you won’t hurt our feelings. We’re doing this to improve the site, so we need to hear your honest reactions. If you have any questions as we go along, just ask them. We may not be able to answer them right away, since we’re interested in how people do when they don’t have someone sitting next to them to help. But if you still have any questions when we’re done, I’ll try to answer them then.

We are going to start with a quick practice on an already available website: [www.cricketaustralia.com.au](http://www.cricketaustralia.com.au)

Practice run task list:

1. Take some time telling us what your general impressions are?
2. What do you want to do first?
3. Please find the contact us button, click on it and read out the phone number if you wanted to call Cricket Australia?
4. Can you go back to the home page?

Researcher:

Okay, it is time to do the test with our website now. Do you have any questions before we start?

ExerciseGuide task list:

1. Please log into the website (hand them an email with login details)
2. Considering this is the first time you logged in, we will give you a minute to take the website in.
3. [Pause 10 seconds]
4. ‘Can you think aloud?’
5. …prompt What are your general impressions?; What caught your eye first?
6. Can you show us what the first thing you might do is?
7. Just from looking at the titles, what modules (topics) seem most interesting to you?
8. Please click into the “getting started” module (if they haven’t already) and watch the “getting started” video
9. Please click on the “Drive Safely” module and complete the questions.
10. From there we would like you to click through until you get to the topic of “Exercise with Metastases.” Can you read this page and the next two at your own pace and in as much detail as you typically would if you were in your own home.
11. Please find the “library,” click on it and find article called “Cancer-related fatigue: Does exercise help or hinder?”
12. Can you please head to the “My Exercise Plan Weeks 1-3” and complete the questions. Please read through the tailored information at your own pace and in as much detail as you typically would if you were in your own home.
13. Please click on the “Tracking module”
14. Do you have any questions about the website?
15. FREE TIME: We have finished completing the set tasks. Are there any other areas of the website that you may want to visit. Please feel free to take time to look at them.
