# Supplementary material for: Usability, Acceptability, and Safety Analysis of a Computer-Tailored Web-Based Exercise Intervention (ExerciseGuide) for Individuals With Metastatic Prostate Cancer: Multi-Methods Laboratory-Based Study
Source: JMIR Cancer. 2021 Jul 28;7(3):e28370. doi: 10.2196/28370 (PMC8367181; doi:10.2196/28370)
Supplement: Multimedia Appendix 2 [file cancer_v7i3e28370_app2.docx]

**Multimedia Appendix 2:** **Movement screening proforma**

Table S1: Upper body exercise movement screening proforma

*Objective scoring criteria (scale): -1 = unsatisfactory with major concerns, 0 = unsatisfactory with major concerns, 1 = satisfactory, 2 = good. Safe is equal to a score of 1 or 2.*

| **Exercise** | **Assessment set up** | **Key points** | **Objective scoring criteria** |
| --- | --- | --- | --- |
| Seated band chest press | Wrap band around the back of your chair. Grip the band around each thumb and make a fist like action. Sit up tall (avoid curling forwards, straining neck or lower back). Extend arms out in front at chest height for 2 seconds and then 2 seconds back to the starting position. | Avoid letting elbows travel back behind your back (which can put extra strain on the front of the shoulders). Keep shoulders relaxed, elbows below underarm height and focus on using chest and arm muscles | Set up based on the provided video-based instruction? |
|  |  |  | Kept their spine in a neutral zone throughout the exercise? |
|  |  |  | Kept trunk vertical (avoided leaning backwards or forwards)? |
|  |  |  | Avoided significant scapular elevation(hitching) throughout the exercise? |
|  |  |  | Kept their shoulder abduction between approximately 40-60 degrees |
|  |  |  | Completed the exercise in a slow, smooth, controlled manner? |
| Incline Push Up | Using a solid object such as a kitchen bench, place your hands slightly wider than shoulder width apart. Start with your arms straight and your body long and straight (you can start on your toes). Slowly lower your chest (and whole body) towards the bench (over the course of 2 seconds) until you gently touch the bench, and then push yourself back to the starting position (taking 2 seconds). | Avoid letting your shoulders scrunch up towards your ears (we want the muscles of the chest/arms rather than your neck muscles to work). Keep your elbows from flaring out towards the side (40-60degrees from your side). Keep your body straight as letting your body sink in the middle can lead to injury. Focus on using chest and arm muscles to create the movement. Abdominals and gluteal muscles can be used to keep the body steady. | Set up based on the provided video-based instruction? |
|  |  |  | Kept their spine in a neutral zone throughout the exercise? |
|  |  |  | Avoided significant scapular elevation (hitching) throughout the exercise? |
|  |  |  | Kept their shoulder abduction between approximately 40-60 degrees? |
|  |  |  | Brought their chest (pectorals) toward hands? |
|  |  |  | Completed the exercise in a slow, smooth, controlled manner? |
| Standing Band Row | Attach a band to an anchor point. Face the band and grip the band around each thumb (make a fist). Step away until there is adequate tension (with your arms outstretched). Stand up tall (avoid curling forwards, arching back or straining neck). Taking 2 seconds, slowly bring fists to underarms by flexing the elbow and pulling the shoulder blades towards the spine, hold for 1 second and then slowly straighten arms and relax shoulder blades. | Avoid letting elbows travel back behind your back (can put extra strain on the front of the shoulders). Keep shoulders relaxed, elbows below underarm height (avoid elbows flaring out). Focus on using chest and arm muscles to create the movement. Abdominals and gluteals can be used to keep the body steady. | Set up based on the provided video-based instruction? |
|  |  |  | Kept their spine in a neutral zone throughout the exercise? |
|  |  |  | Avoided significant scapular elevation (hitching) throughout the exercise? |
|  |  |  | Exhibited retraction and protraction of the scapula during the exercise |
|  |  |  | Minimised abduction of the elbow (avoidance of elbow flare)? |
|  |  |  | Completed the exercise in a slow, smooth, controlled manner? |
| Seated Band Row  Seated Band Row (cont.) | Attach band to an anchor point. Sit on the edge of the chair, with a tall posture (avoid curling forwards, arching back or straining neck). Face the band and grip the band around each thumb (make a fist) ensuring there is adequate tension (arms outstretched). Taking 2 seconds, slowly bring fists to underarms by flexing the elbow and pulling the shoulder blades towards the spine, hold for 1 second and then slowly straighten arms and relax shoulder blades. | Avoid rocking the upper body to generate momentum. Keep shoulders relaxed, elbows below underarm height (avoid elbows flaring out). Focus on using mid-back and arm muscles to create the movement and abdominals to keep trunk steady | Set up based on the provided video-based instruction (see booklet)? |
|  |  |  | Kept their spine in a neutral zone throughout the exercise? |
|  |  |  | Avoided significant scapular elevation (hitching) throughout the exercise? |
|  |  |  | Exhibited retraction and protraction of the scapula during the exercise? |
|  |  |  | Kept trunk vertical (avoided leaning backwards or forwards)? |
|  |  |  | Minimised abduction of the elbow (Avoidance of elbow flare) |
|  |  |  | Completed the exercise in a slow, smooth, controlled manner? |
| Standing Band Bicep Curl | Sit on the edge of the chair, with a tall posture (avoid curling forwards, arching back or straining neck). Wrap theraband around hands (making a fist) and place the band under your feet (keeping feet flat on the ground). Taking 2 seconds, slowly bring fists to shoulders (palms facing shoulders) by flexing the elbow, hold for 1 second and then slowly lower again till arms are almost straight | Keep elbows touching the side of your body throughout the whole movement. Avoid rocking the upper body to generate momentum. Keep shoulders relaxed and chest up tall. Focus on using bicep muscles to create the movement and abdominals to keep trunk steady | Set up based on the provided video-based instruction (see booklet)? |
|  |  |  | Kept their spine in a neutral zone throughout the exercise? |
|  |  |  | Kept a neutral scapular position (minimal hitching, forward shoulder) during exercise? |
|  |  |  | Avoided significant elbow deviation from the start position (both forward and lateral) |
|  |  |  | Complete the full range of motion (approximately 0°-110°) |
|  |  |  | Completed the exercise in a slow, smooth, controlled manner? |
| Seated Band Bicep Curl | Sit on the edge of the chair, with a tall posture (avoid curling forwards, arching back or straining neck). Wrap theraband around hands (making a fist) and place band under your feet (keeping feet flat on the ground). Taking 2 seconds, slowly bring fists to shoulders (palms facing shoulders) by flexing the elbow, hold for 1 second and then slowly lower again till arms are almost straight | Keep elbows touching the side of your body throughout the whole movement. Avoid rocking the upper body to generate momentum. Keep shoulders relaxed and chest up tall. Focus on using bicep muscles to create the movement and abdominals to keep trunk steady | Set up based on the provided video-based instruction (see booklet)? |
|  |  |  | Kept their spine in a neutral zone throughout the exercise? |
|  |  |  | Kept a neutral scapular position (minimal hitching, forward shoulder) during exercise? |
|  |  |  | Kept trunk vertical (avoided leaning backwards or forwards)? |
|  |  |  | Avoided significant elbow deviation from the start position (both forward and lateral) |
|  |  |  | Complete the full range of motion (approximately 0°-140°) |
|  |  |  | Completed the exercise in a slow, smooth, controlled manner? |
| Seated Band Triceps Extension | Attach band to an anchor point at about head height. Sit on the edge of the chair, with a tall posture (avoid curling forwards, arching back or straining neck). Face the band and grip the band around each thumb (make a fist) ensuring there is adequate tension. Start with your arms bent by your sides and whilst keeping your elbows touching your side, slowly extend your arms till they are straight (taking 2 seconds). Pause for 1 second and then slowly bring your arms to the starting position (bent by sides). Pause for 1 second before starting your next repetition | Focus on using shoulder and arm (triceps) muscles to create the movement. Abdominals can be used to keep the body steady. Avoid letting your shoulders scrunch up towards your ears (we want the muscles of the arms and not neck to work). Keep elbows in the same spot throughout the action, otherwise we are not targeting the appropriate muscles. | Set up based on the provided video-based instruction? |
|  |  |  | Kept their spine in a neutral zone throughout the exercise? |
|  |  |  | Kept a neutral scapular position (minimal hitching, forward shoulder) during exercise? |
|  |  |  | Kept trunk vertical (avoided leaning backwards or forwards)? |
|  |  |  | Avoided significant elbow deviation from the start position (both forward and lateral) |
|  |  |  | Complete the full range of motion (approximately 110°-0°) |
|  |  |  | Completed the exercise in a slow, smooth, controlled manner? |

Table S1: Upper body exercise movement screening proforma (continued)

*Objective scoring criteria (scale): -1 = unsatisfactory with major concerns, 0 = unsatisfactory with major concerns, 1 = satisfactory, 2 = good. Safe is equal to a score of 1 or 2.*

Table S2: Trunk-focused exercise movement screening proforma

*Objective scoring criteria (scale): -1 = unsatisfactory with major concerns, 0 = unsatisfactory with major concerns, 1 = satisfactory, 2 = good. Safe is equal to a score of 1 or 2.*

| **Exercise** | **Assessment set up** | **Key points** | **Objective scoring criteria** |
| --- | --- | --- | --- |
| Seated March | Sit on the edge of the chair, with a tall posture (avoid curling forwards, arching back or straining neck). Ensure feet are flat on the floor. Over the course of 2 seconds, slowly raise knee up as high as you can without curling your spine or leaning back and then slowly straighten your leg. Hold for 1 second and very slowly lower. Alternate legs each repetition. | Aim to keep your trunk vertical and avoid shifting too much weight when alternating sides. The muscles of the trunk should be used to keep your trunk still and the muscles around the front of the hip. Keep shoulders and neck relaxed | Set up based on the provided video-based instruction? |
|  |  |  | Kept their spine in a neutral zone throughout the exercise? |
|  |  |  | Kept trunk vertical (avoided leaning backwards or forwards)? |
|  |  |  | Kept shoulders and neck relaxed (not co-contracting to assist movement) |
|  |  |  | Completed the exercise in a slow, smooth, controlled manner? |
| Leg Fallout | Lie on the ground on you back, with your knees bent and feet flat on the ground. Ensure your body weight is evenly spread over the back of your hips. Focus on keeping your hips touching the ground at all times, as you slowly control (over 2 seconds) one knee falling out towards the ground to the side of you. The aim is to go as far as you can before your opposite hip moves (you may want to keep your hands on your hips to feel any movement). When you cannot go any further, slowly bring the leg back to the starting position. Alternate sides each repetition. | The deeper muscles of the abdominals should be used to keep your trunk/hips still as you move. Breathe as normally as possible. Keep shoulders and neck relaxed. | Set up based on the provided video-based instruction (see booklet)? |
|  |  |  | Kept their spine in a neutral zone throughout the exercise? |
|  |  |  | Kept their pelvis level throughout the movement (watch for lateral movement) |
|  |  |  | Kept shoulders and neck relaxed (not co-contracting to assist movement) |
|  |  |  | Completed the exercise in a slow, smooth, controlled manner? |
| Single Leg Lift | Lie on the ground on you back, with your knees bent and feet flat on the ground. Ensure your body weight is evenly spread over the back of your hips and you have a gentle curve in you lower back (neutral spine). Focus on keeping your hips in this position over the course of the repetition. The aim is to very slowly (2 seconds) lift your leg up to approximately 90 degrees and hold for 1 second before slowly lowering to the starting position. Alternate legs (but avoid rocking through your pelvis, spine or rib cage) | The deeper muscles of the abdominals should be used to keep your trunk/hips still as you move. We want to avoid a large doming of your outer abdominals (a big puff up) as this means you may not have engaged your deeper muscles. You can have your hands resting on your stomach or hips to help you feel any doming or rocking. Breathe as normally as possible. Keep shoulders and neck relaxed. | Set up based on the provided video-based instruction? |
|  |  |  | Kept their spine in a neutral zone throughout the exercise? |
|  |  |  | Avoided abdominal doming (significant activation of rectus abdominis)? |
|  |  |  | Kept show3ulders and neck relaxed (not co-contracting to assist movement)? |
|  |  |  | Completed the exercise in a slow, smooth, controlled manner? |
| Single Leg Lift with Extension | Lie on the ground on you back, with your knees bent and feet flat on the ground. Ensure your body weight is evenly spread over the back of your hips and you have a gentle curve in you lower back (neutral spine). Focus on keeping your hips in this position over the course of the repetition. The aim is to very slowly (2 seconds) lift your leg up to approximately 90 degrees, hold for 1 second before slowly lowering the leg out straight. This will add extra load onto the working muscles (avoid arching through the spine). Slowly bring the leg back to the 90 degree position and then return to the starting position. Alternate legs (but avoid rocking through your pelvis, spine or rib cage). | The deeper muscles of the abdominals should be used to keep your trunk/hips still as you move. We want to avoid a large doming of your outer abdominals (a big puff up) as this means you may not have engaged your deeper muscles. Breathe as normally as possible and aim to keep the shoulders and neck relaxed. | Set up based on the provided video-based instruction (see booklet)? |
|  |  |  | Kept their spine in a neutral zone throughout the exercise? |
|  |  |  | Avoided abdominal doming (significant activation of rectus abdominis)? |
|  |  |  | Kept shoulders and neck relaxed (not co-contracting to assist movement)? |
|  |  |  | Completed the exercise in a slow, smooth, controlled manner? |
| Double Leg Lift | Lie on the ground on you back, with your knees bent and feet flat on the ground. Ensure your body weight is evenly spread over the back of your hips and you have a gentle curve in you lower back (neutral spine). Focus on keeping your hips in this position over the course of the whole exercise. The aim is to very slowly (2 seconds) lift your leg up to approximately 90 degrees, hold for 1 second before slowly bringing the other leg up to the same position. This will add extra load onto the working muscles (avoid arching through the spine). Slowly bring the second leg back to the ground and then return the first leg to the starting position. Alternate legs (but avoid rocking through your pelvis, spine or rib cage). | The deeper muscles of the abdominals should be used to keep your trunk/hips still as you move. We want to avoid a large doming of your outer abdominals (a big puff up) as this means you may not have engaged your deeper muscles. Breathe as normally as possible. Keep shoulders and neck relaxed. | Set up based on the provided video-based instruction (see booklet)? |
|  |  |  | Kept their spine in a neutral zone throughout the exercise? |
|  |  |  | Avoided abdominal doming (significant activation of rectus abdominis)? |
|  |  |  | Kept shoulders and neck relaxed (not co-contracting to assist movement)? |
|  |  |  | Completed the exercise in a slow, smooth, controlled manner? |
| Double Leg Hip Lift | Lie on the ground on you back, with your knees bent and feet flat on the ground. Ensure your body weight is evenly spread over the back of your hips and you have a gentle curve in you lower back (neutral spine). Focus on keeping your hips in this position over the course of the whole exercise. The aim is to very slowly (2 seconds) lift your hips off the ground using your gluteal muscles as high as you can without arching your spine. Hold for 1 second at the top and then slowly (2 seconds) bring your hips back onto the ground evenly. | The gluteal muscles should be used to lift your hips and the deeper muscles of the abdominals should be used to keep your trunk/hips still as you move. Breathe as normally as possible. Keep shoulders and neck relaxed. | Set up based on the provided video-based instruction (see booklet)? |
|  |  |  | Kept their spine in a neutral zone throughout the exercise? |
|  |  |  | Kept their pelvis level throughout the movement? |
|  |  |  | Extended hips to an approximate full range (0-15 degrees hip flexion)? |
|  |  |  | Completed the exercise in a slow, smooth, controlled manner? |
| All Fours With Single Leg Extension | Position yourself on hands and knees, with your body weight is evenly spread evenly and you have a gentle curve in you lower back (neutral spine). The aim is to very slowly (2 seconds) lift one leg/knee off the ground, without shifting your body weight, arching spine or tipping hips (you want to imagine your back is a table which has a glass of water on it that you do not want to spill). Hold for 1 second when you have reach as far as your leg can comfortably lift and then slowly (2 seconds) bring your leg back to the ground. Alternate which leg you lift each repetition. | The deeper muscles of the abdominals should be used to keep your trunk/hips still as you move and your glute muscles are used to help lift the leg. Avoid collapsing through the shoulders/mid-back and chest – keep your chest tall. Go slow as the aim is to keep balance throughout the entire movement. Breathe as normally as possible. | Set up based on the provided video-based instruction (see booklet)? |
|  |  |  | Kept their spine in a neutral zone throughout the exercise? |
|  |  |  | Kept their scapula in a neutral scapular position (minimal hitching/winging/tilting) during the exercise |
|  |  |  | Do they maintain balance while the leg is off the ground |
|  |  |  | Kept a level pelvis throughout the movement |
|  |  |  | Completed the exercise in a slow, smooth, controlled manner? |

Table S2: Trunk-focused exercise movement screening proforma

(continued)

*Objective scoring criteria (scale): -1 = unsatisfactory with major concerns, 0 = unsatisfactory with major concerns, 1 = satisfactory, 2 = good. Safe is equal to a score of 1 or 2.*

Table S3: Lower body exercise movement screening proforma

*Objective scoring criteria (scale): -1 = unsatisfactory with major concerns, 0 = unsatisfactory with major concerns, 1 = satisfactory, 2 = good. Safe is equal to a score of 1 or 2.*

| **Exercise** | **Assessment set up** | **Key points** | **Objective scoring criteria** |
| --- | --- | --- | --- |
| Sit to stand | Start seated with feet a little wider than shoulder-width apart and cross your arms. Initiate the movement by leaning through the hips and driving through the bottom to stand up. Focus on keeping your spine relatively long and straight (bend from hips, not lower back). Keep heels on the ground and spread your bodyweight evenly between the heel and ball of each foot. Once you are standing up fully, very slowly (over 2 seconds) unlock the hips, bringing them back towards the seat and allow you knees to bend to continue the movement. Once your bottom is touching the seat, complete the next repetition. | The gluteal muscles are used to stand up and the quadriceps muscles are used to lower yourself towards the seat. Keep the knees, ankles in line with each other (they should run in two lines like train tracks), we want to avoid the knees collapsing inwards. Breathe as normally as possible. Avoid rounding the shoulders or back (we are only moving through the hips, knees and ankles. | Set up based on the provided video-based instruction (see booklet)? |
|  |  |  | Kept their spine in a neutral zone throughout the exercise? |
|  |  |  | Kept their pelvis parallel to the ground throughout the exercise? |
|  |  |  | Kept their knees travelling in the same direction as their hips and toes (no excessive varus or valgus movement)? |
|  |  |  | Kept both feet in full contact with the ground (heels and balls of feet)? |
|  |  |  | Completed the exercise in a slow, smooth, controlled manner? |
| Partial (1/4) Squat | Stand tall with feet a little wider than shoulder-width apart and cross your arms. Initiate the movement by unlocking the hips, slightly bringing them back towards a seat and allow you knees to bend to continue the movement. Focus on keeping your spine relatively long and straight (bend from hips, not lower back). As you bottom goes back, allow your knees to travel forwards, so your centre of gravity remains through your ankles. Keep heels on the ground and spread your bodyweight evenly between the heel and ball of each foot. Continue to squat down until your thighs are at an angle of 45degrees from the floor (halfway from standing to sitting). Pause for 1 second before standing up tall. | The quadriceps muscles are used to lower yourself towards the seat and gluteal muscles are used to stand up. Keep the knees, ankles in line with each other (they should run in two lines like train tracks), we want to avoid the knees collapsing inwards. Breathe as normally as possible. Avoid rounding the shoulders or back (we are only moving through the hips, knees and ankles. | Set up based on the provided video-based instruction (see booklet)? |
|  |  |  | Kept their spine in a neutral zone throughout the exercise? |
|  |  |  | Kept their pelvis parallel to the ground throughout the exercise? |
|  |  |  | Kept their knees travelling in the same direction as their hips and toes (no excessive varus or valgus movement)? |
|  |  |  | Kept both feet in full contact with the ground (heels and balls of feet)? |
|  |  |  | Maintained a similar hip flexion angle in comparison to dorsiflexion angle? |
|  |  |  | Achieved a quarter squat depth? |
|  |  |  | Completed the exercise in a slow, smooth, controlled manner? |
| Squat (1/2) | Stand tall with feet a little wider than shoulder-width apart and cross your arms. Initiate the movement by unlocking the hips, slightly bringing them back towards a seat and allow you knees to bend to continue the movement. Focus on keeping your spine relatively long and straight (bend from hips, not lower back). As you bottom goes back, allow your knees to travel forwards, so your centre of gravity remains through your ankles. Keep heels on the ground and spread your bodyweight evenly between the heel and ball of each foot. Continue to squat down until your thighs are at an angle of about 90 degrees from the floor, just gently touching the front of a seat. Pause for 1 second before standing up tall. | The quadriceps muscles are used to lower yourself towards the seat and gluteal muscles are used to stand up. Keep the knees, ankles in line with each other (they should run in two lines like train tracks), we want to avoid the knees collapsing inwards. Breathe as normally as possible. Avoid rounding the shoulders or back (we are only moving through the hips, knees and ankles. | Set up based on the provided video-based instruction (see booklet)? |
|  |  |  | Kept their spine in a neutral zone throughout the exercise? |
|  |  |  | Kept their pelvis parallel to the ground throughout the exercise? |
|  |  |  | Kept their knees travelling in the same direction as their hips and toes (no excessive varus or valgus movement)? |
|  |  |  | Kept both feet in full contact with the ground (heels and balls of feet)? |
|  |  |  | Maintained a similar hip flexion angle in comparison to dorsiflexion angle? |
|  |  |  | Achieved a hip flexion angle of approximately 90 degrees (half squat depth)? |
|  |  |  | Completed the exercise in a slow, smooth, controlled manner? |
| Seated Band Knee Extension | Sit on the edge of the chair, with a tall posture (avoid curling forwards, arching back or straining neck). Begin by looping the centre of the band around the ankle of your exercising leg. Bring the ends of the band underneath the foot of the opposite leg (which is pulled back as far as it can go) to stabilize and grasp the ends by your knee. Slowly extend your leg so your knee is straight against the band over a count of 3 seconds. Hold for 1 second and then slowly (3 seconds) bring your exercising leg back to the starting position. | Ensure the band is tight enough to challenge the muscles of the thigh. Keep the arm/shoulder/neck of the hand holding the band as relaxed as possible. Focus on using the muscles of the thigh and not the knee joint itself. Make sure you do not rock forward or back, this will make the exercise easier. | Set up based on the provided video-based instruction (see booklet)? |
|  |  |  | Kept their spine in a neutral zone throughout the exercise? |
|  |  |  | Kept their hip, knee and foot remain aligned throughout the movement? |
|  |  |  | Achieved a full range of motion (0‐90°) without hyperextension? |
|  |  |  | Kept trunk vertical (avoided leaning backwards or forwards)? |
|  |  |  | Completed the exercise in a slow, smooth, controlled manner? |
| Seated Band Hamstring Curl | Sit on the edge of the chair, with a tall posture (avoid curling forwards, arching back or straining neck). Begin by looping the centre of the band around the ankle of your exercising leg. Bring the ends of the band underneath the foot of the opposite leg (which is as far forward as it can go) to stabilize and grasp the ends by your knee. Slowly pull your heel back towards your bottom as far as you can over a count of 3 seconds. You should feel this in the back of your thigh. Hold for 1 second and then slowly (3 seconds) bring your leg back to the starting position. | Ensure the band is tight enough to challenge the muscles of the back of the thigh. Keep the arm/shoulder/neck of the hand holding the band as relaxed as possible. Focus on using the muscles of the thigh and not the knee or ankle joint itself. Make sure you do not rock forward or back, this will make the exercise easier. | Set up based on the provided video-based instruction (see booklet)? |
|  |  |  | Kept their spine in a neutral zone throughout the exercise? |
|  |  |  | Kept their hip, knee and foot remain aligned throughout the movement? |
|  |  |  | Kept trunk vertical (avoided leaning backwards or forwards)? |
|  |  |  | Completed the exercise in a slow, smooth, controlled manner? |
| Standing Calf Raise | Start standing in front of a stable object or wall with feet shoulder-width apart.  Lift your heels off the ground as high as you can, spreading weight evenly through ball of big toe and outside toe. Hold at the top for 1 second. Very slowly lower yourself to the ground over the course of two seconds before repeating the exercise again. | The calf muscles are the main muscles working to lift your heels off of the ground. Keep the knees, ankles in line with each other (they should run in two lines like train tracks). Keep yourself standing as tall as possible, avoid bending back or hips. | Set up based on the provided video-based instruction (see booklet)? |
|  |  |  | Kept their spine in a neutral zone throughout the exercise? |
|  |  |  | Kept their hips and knees in vertical alignment with the shoulders and ankles? |
|  |  |  | Appeared to keep their foot neutral (even weight distribution on all metatarsals)? |
|  |  |  | Kept their full range of motion for the full set? |
|  |  |  | Completed the exercise in a slow, smooth, controlled manner? |

Table S3: Lower body exercise movement screening proforma (continued)

(continued)

*Objective scoring criteria (scale): -1 = unsatisfactory with major concerns, 0 = unsatisfactory with major concerns, 1 = satisfactory, 2 = good. Safe is equal to a score of 1 or 2.*
