# Supplementary material for: Usability, Acceptability, and Safety Analysis of a Computer-Tailored Web-Based Exercise Intervention (ExerciseGuide) for Individuals With Metastatic Prostate Cancer: Multi-Methods Laboratory-Based Study
Source: JMIR Cancer. 2021 Jul 28;7(3):e28370. doi: 10.2196/28370 (PMC8367181; doi:10.2196/28370)
Supplement: Multimedia Appendix 3 [file cancer_v7i3e28370_app3.docx]

**Multimedia Appendix 3. Semi-structured interview guide**

Table S4: Semi-structured interview guide

| **Topic** | **Question/s** |
| --- | --- |
| Website | 1. What things did you like about the website?  2. What didn’t you like about the website? |
| Exercise prescription | 3. Can you tell me about what you thought about the exercises prescribed? Easy/hard. Do you think you could do them at home without someone helping?  4. How did you find the videos? |
| ExerciseGuide program | 1. Can you provide some feedback on the pros of this program? 2. Can you provide some feedback on the cons of this program? 3. a) Would you like to have access to an exercise professional (via phone or video conference) to aid accountability and help in other aspects of the program? *   b) How often would you like the exercise professional to contact you?   1. What could we do to improve the program in the future? |
| **Please note question 7 was added to the interview after the first iterative changes to the ExerciseGuide program after feedback from previous participants.* | |
