# Supplementary material for: Usability, Acceptability, and Safety Analysis of a Computer-Tailored Web-Based Exercise Intervention (ExerciseGuide) for Individuals With Metastatic Prostate Cancer: Multi-Methods Laboratory-Based Study
Source: JMIR Cancer. 2021 Jul 28;7(3):e28370. doi: 10.2196/28370 (PMC8367181; doi:10.2196/28370)
Supplement: Multimedia Appendix 4 [file cancer_v7i3e28370_app4.docx]

**Multimedia Appendix 4: Think-aloud Modifications**

Table S5: Usability testing feedback and revisions made

| **Cycle** | **Topic** | **Observation feedback from users** | **Revisions made** |
| --- | --- | --- | --- |
| One  (n=5) | Navigation | A navigation tutorial video would be helpful | A navigation tutorial video was added |
|  | Design | Text too small | Text size increased |
|  |  | Modules were too long | Content tailored further. Dot points. Additional information moved to the library. |
|  | Content | Medical terminology is confusing | Reduced medical terminology. Links added for additional information in the library. Matched metastases locations in questions to patient information form. |
|  |  | Discuss maintenance of health, not just progressions. Some participants do not want to progress, just avoid deconditioning | Information revised to reduce the emphasis on progressing and increase information on ‘minimum dose’ exercise for maintenance. |
|  | Exercise prescription | Modifications of prescriptions for those already meeting aerobic or resistance targets. | Tailored information to explain how participants integrate the Exercise Guide program into their current exercise schedule. |
|  |  | A maximum of ten exercises is too many | Maximum of eight exercises prescribed |
|  | Program | Additional of further support to aid adherence | Addition of a telehealth component to intervention. |
| Two  (n=6) | Navigation | Unable to use embedded videos easily (both starting and exiting the video) | A video tutorial was made to explain typical issues encountered (how to watch and then exit an embedded video. |
|  |  | Navigation videos need to be slower | Re-create videos with increased duration to explain the navigation. |
|  | Design | Ensure images are not all Anglocentric | Change some images to ensure |
|  |  | Ensure consistency of style and grammar | Changes made to style and grammar |
|  |  | Two modules asked very similar questions for tailoring. Reduce question double-ups. | Moved important tailoring questions (i.e. metastasis location) to the “getting started” module to ensure completion – this data is drawn into other tailoring algorithms. |
|  | Exercise Prescription | Would like other methods of exercise prescription (if safe) | Other methods (i.e.: rowing and swimming) were prescribed for individuals without bone metastases. Walking in water was added for individuals with bone metastases. |
|  |  | Addition of stretching exercises | Tailored stretching exercises to resistance training exercises prescribed |
|  | Program | More information needed to address distress and hot flushes | Additional information added into the Exercise plus and where else can I get help modules around these two areas. |
