# Supplementary material for: Usability, Acceptability, and Safety Analysis of a Computer-Tailored Web-Based Exercise Intervention (ExerciseGuide) for Individuals With Metastatic Prostate Cancer: Multi-Methods Laboratory-Based Study
Source: JMIR Cancer. 2021 Jul 28;7(3):e28370. doi: 10.2196/28370 (PMC8367181; doi:10.2196/28370)
Supplement: Multimedia Appendix 5 [file cancer_v7i3e28370_app5.docx]

**Multimedia Appendix 5: *ExerciseGuide* intervention-specific changes pre- and post usability study.**

Table S6: Intervention-specific changes pre and post usability and acceptability study.

| **Program** | **Specific** | **Pre-usability study** | **Post-usability study** |
| --- | --- | --- | --- |
| Information modules | Getting started | Text to explain website navigation | Text and video to explain website navigation  Additional tailoring questions (metastases location) |
|  | Exercise benefits | Text and images to explain benefits of exercise  Tailored information (text/images) based on benefits of exercise on:   1. Reduced muscular strength 2. Fatigue 3. Anxiety and or depression 4. Bone pain 5. Sleep 6. Incontinence 7. Sexual function 8. Balance | Text and images to explain benefits of exercise  Tailored information (text/images) based on benefits of exercise on:   1. Reduced muscular strength 2. Fatigue 3. Anxiety and or depression 4. Bone pain 5. Sleep 6. Incontinence 7. Sexual function 8. Balance |
|  | Drive safely | Tailored information (text/images) on:   1. Exercise safety whilst undergoing treatments 2. Exercise safety based on bone metastases location/s 3. Aerobic exercise safety 4. Exercise safely with treatment side effects 5. When should exercises be paused or stopped 6. Exercising safely with other medical conditions | Tailored information (text/images) on:   1. Exercise safety whilst undergoing treatments 2. Exercise safety based on bone metastases location/s 3. Aerobic exercise safety 4. Exercise safely with treatment side effects 5. When should exercises be paused or stopped 6. Exercising safely with other medical conditions 7. Monitoring exercise |
|  | Making it last | Tailored information (text/images) on:   1. Factors that influence exercise 2. How to improve confidence to exercise 3. How to plan 4. Motivation 5. Habits | Tailored information (text/images) on:   1. Factors that influence exercise 2. How to improve confidence to exercise 3. How to plan 4. Motivation 5. Habits |
|  | Exercise Plus | Tailored information (text/images) on:   1. Nutrition 2. Sedentary behaviours 3. Alcohol 4. Sleep | Tailored information (text/images) on:   1. Nutrition 2. Sedentary behaviours 3. Alcohol 4. Sleep 5. Distress 6. Hot flushes |
|  | Where else can I get help | Tailored information (text/images) on:   1. General information for advanced prostate cancer 2. Further exercise resources support 3. Further diet and nutrition support 4. Further information for symptom support 5. Further sleep support 6. Location based support 7. Resources for Aboriginal and Torres Strait Islander support 8. Resources for individuals with English as a second language 9. Resources for individuals in the LGBTIQ community | Tailored information (text/images) on:   1. General information for advanced prostate cancer 2. Further exercise resources support 3. Further diet and nutrition support 4. Further information for symptom support 5. Further sleep support 6. Location based support 7. Resources for Aboriginal and Torres Strait Islander support 8. Resources for individuals with English as a second language 9. Resources for individuals in the LGBTIQ community 10. Further information to assist with distress |
| Exercise Prescription modules | Duration | 8-week intervention | 8-week intervention |
|  | Resistance exercise prescription | Maximum of ten exercises prescribed per program | Maximum of eight exercises prescribed per program |
|  |  | Mode: Body weight and resistance exercises | Mode: Body weight and resistance exercises |
|  |  | Frequency: Two to three sessions per week. | Frequency: Two to three sessions per week but tailored to encourage participants who are already completing two or more sessions to fit the prescription in to current routine. |
|  |  | Intensity: 6-7 out of 10 | Intensity: 6-7 out of 10 |
|  | Aerobic exercise prescription | Modes: Walking and cycling | Modes: Walking, cycling and other methods (i.e.: rowing and swimming) were prescribed for individuals without bone metastases. Walking in water was added for individuals with bone metastases. |
|  |  | Frequency: Two to five sessions per week. | Frequency: Two to five sessions per week but tailored to encourage participants who are already completing two or more sessions to fit the prescription in to current routine. |
|  |  | Intensity: 6-7 out of 10 | Intensity: 6-7 out of 10 |
|  | Flexibility exercise prescription | None prescribed | Program prescribed based upon resistance training muscle groups used |
|  | Other | Exercise prescription tailored to exercise volume or intensity progressions. | Exercise prescription tailored to either exercise volume or intensity progressions or maintenance of health. |
| Support | Telehealth | No telehealth included | Three telehealth consults (via real-time teleconferencing or phone calls) at week 1, week 4 and week 8. Approximately 20 minutes in duration. |
|  | How are you tracking | Includes personalised information of:   1. Participants weekly activity summary 2. Goal progression tracker 3. Symptom tracker 4. Confidence tracker | Includes personalised information of:   1. Participants weekly activity summary 2. Goal progression tracker 3. Symptom tracker 4. Confidence tracker |

A detailed explanation of the ExerciseGuide intervention after the completion of this study can be found at: Evans HE, Forbes CC, Galvão DA, Vandelanotte C, Newton RU, Wittert G, Chambers S, Vincent AD, Kichenadasse G, Brook N, Girard D. Evaluating a web-and telephone-based personalised exercise intervention for individuals living with metastatic prostate cancer (ExerciseGuide): protocol for a pilot randomised controlled trial. Pilot and feasibility studies. 2021;7(1):1-6.
