# Supplementary material for: Usability, Acceptability, and Safety Analysis of a Computer-Tailored Web-Based Exercise Intervention (ExerciseGuide) for Individuals With Metastatic Prostate Cancer: Multi-Methods Laboratory-Based Study
Source: JMIR Cancer. 2021 Jul 28;7(3):e28370. doi: 10.2196/28370 (PMC8367181; doi:10.2196/28370)
Supplement: Multimedia Appendix 6 [file cancer_v7i3e28370_app6.docx]

**Multimedia Appendix 6:** **Movement screening scores and ICC interrater reliability**

Table S7. Movement screening scores and intraclass correlation to estimate interrater reliability

| **Region** | **Exercise** | **Number of screening items per exercise** | **Possible range of scores** | **Safety cut point** | **Expert movement screening scores** | | | | | | | | **Overall score**  M (SD) | **ICC** |
| --- | --- | --- | --- | --- | --- | --- | --- | --- | --- | --- | --- | --- | --- | --- |
|  |  |  |  |  | **Expert 1**  M (SD) | **Expert 2**  M (SD) | | **Expert 3**  M (SD) | | **Expert 4**  M (SD) | | **Expert 5**  M (SD) |  |  |
| Upper body exercises | Seated Bicep Curl (N=4) | 7 | 7-28 | <14 | 22.0 (2.9) | | 19.0 (7.7) | | 25.3 (2.2) | | 21.0 (3.7) | 26.5 (1.0) | 22.8 (4.7) | 0.197 |
|  | Standing Bicep Curl (N=6) | 6 | 6-24 | <12 | 22.5 (1.4) | | 17.7 (2.4) | | 22.7 (1.4) | | 19.3 (1.9) | 22.7 (1.4) | 21.0 (2.7) | 0.000 |
|  | Seated Triceps Extension (N=9) | 6 | 6-24 | <12 | 16.7 (3.2) | | 10.7 (4.7) | | 21.4 (1.2) | | 15.0 (4.7) | 22.6 (1.0) | 17.4 (5.4) | 0.053 |
|  | Incline Push Up (N=3) | 6 | 6-24 | <12 | 19.3 (4.6) | | 18.0 (6.9) | | 22.7 (1.2) | | 18.0 (3.0) | 22.7 (1.5) | 20.1 (4.1) | 0.103 |
|  | Seated Chest Press (N=2) | 6 | 6-24 | <12 | 23.5 (0.7) | | 16.5 (0.7) | | 24.0 (0.0) | | 20.5 (2.1) | 24.0 (0.0) | 21.7 (3.2) | 0.000 |
|  | Seated Row (N=3) | 7 | 7-28 | <14 | 22.7 (2.3) | | 21.3 (5.1) | | 27.0 (1.7) | | 16.7 (3.1) | 27.3 (1.2) | 23.0 (4.8) | 0.000 |
|  | Standing Row (N=1)* | 6 | 6-24 | <12 | 19.3 (4.6) | | 18.0 (6.9) | | 22.7 (1.2) | | 18.0 (3.0) | 22.7 (1.5) | 21.4 (3.6) | N/A |
| Trunk exercises | Seated March with Leg Extension (N=4) | 5 | 5-20 | <10 | 17.3 (2.2) | | 11.8 (2.8) | | 19.5 (1.0) | | 16.3 (3.0) | 19.8 (0.5) | 16.9 (3.5) | <0.001 |
|  | Leg Fallout (N=5) | 5 | 5-20 | <10 | 18.8 (2.2) | | 16.4 (1.8) | | 18.0 (4.5) | | 16.2 (4.3) | 17.8 (4.4) | 17.4 (3.5) | 0.592 |
|  | Single Leg Lift (N=3) | 5 | 5-20 | <10 | 19.7 (0.6) | | 17.3 (2.1) | | 20.0 (0.0) | | 18.3 (1.5) | 20.0 (0.0) | 19.1 (1.5) | 0.068 |
|  | Double Leg Lift (N=2) | 5 | 5-20 | <10 | 19.7 (0.6) | | 16.0 (3.0) | | 19.7 (0.6) | | 16.7 (1.2) | 19.7 (0.6) | 18.3 (2.1) | 0.000 |
|  | Double Leg Hip Lift (N=1)* | 5 | 5-20 | <10 | 19.0 (0.0) | | 19.0 (0.0) | | 20.0 (0.0) | | 20.0 (0.0) | 20.0 (0.0) | 19.6 (0.6) | N/A |
|  | All Fours with Single Leg Extension (N=2) | 6 | 6-24 | <12 | 23.0 (0.0) | | 15.5 (5.0) | | 19.5 (0.7) | | 21.0 (1.4) | 22.5 (0.7) | 20.3 (3.3) | <0.001 |
| Lower body exercises | Sit to stand (N=1)* | 6 | 6-24 | <12 | 22.0 (0.0) | | 21.0 (0.0) | | 24.0 (0.0) | | 23.0 (0.0) | 24.0 (0.0) | 22.8 (1.3) | N/A |
|  | Squat (N=3) | 8 | 8-32 | <16 | 28.7 (1.2) | | 27.0 (1.7) | | 31.0 (1.7) | | 30.3 (2.1) | 31.7 (0.6) | 29.7 (3.0) | 0.000 |
|  | Seated Knee Extension (N=6) | 6 | 6-24 | <12 | 20.7 (1.1) | | 16.5 (2.9) | | 23.2 (1.2) | | 19.2 (1.9) | 23.2 (0.8) | 20.5 (3.0) | 0.000 |
|  | Seated Hamstring Curl (N=6) | 5 | 5-20 | <12 | 21.5 (1.4) | | 21.3 (1.2) | | 23.8 (0.4) | | 19.7 (1.2) | 24.0 (0.0) | 22.1 (1.9) | 0.000 |
|  | Standing calf raise (N=11) | 6 | 6-24 | <12 | 21.5 (1.4) | | 21.2 (1.5) | | 23.8 (0.4) | | 19.8 (1.8) | 22.9 (3.0) | 21.9 (2.2) | <0.001 |

*ICC not determined due to single sample.

An exercise was deemed safe if, on average, it was scored as satisfactory or good, with the cut point indicating this score.

Original objective scoring criteria (scale): -1 = unsatisfactory with major concerns, 0 = unsatisfactory with major concerns, 1 = satisfactory, 2 = good.

For statistical purposes, scores were then transformed into positive scores: 1 = unsatisfactory with major concerns, 2 = unsatisfactory with major concerns, 3 = satisfactory, 4 = good.
